# Death place and palliative outcome indicators in patients under palliative home care service: an observational study

**DOI:** 10.1186/s12904-023-01167-8

**Published:** 2023-04-19

**Authors:** Pei-Jung Chang, Cheng-Fu Lin, Ya-Huei Juang, Jui-Yu Chiu, Lung-Chun Lee, Shih-Yi Lin, Yu-Hui Huang

**Affiliations:** 1 Home Health Care Agency, Taichung Veterans General Hospital, Taichung, 40705 Taiwan; 2 Department of Nursing, Taichung Veterans General Hospital, Taichung, 40705 Taiwan; 3 Center for Geriatrics & Gerontology, Taichung Veterans General Hospital, Taichung, 40705 Taiwan; 4 Division of Occupational Medicine, Department of Emergency, Taichung Veterans General Hospital, Taichung, 40705 Taiwan; 5 Research Center for Geriatrics and Gerontology, College of Medicine, National Chung Hsing University, Taichung, 40200 Taiwan; 6 Department of Post-Baccalaureate Medicine, College of Medicine, National Chung Hsing University, Taichung, 40200 Taiwan; 7 Department of Family Medicine, Taichung Veterans General Hospital, Taichung, 40705 Taiwan; 8 Division of Endocrinology and Metabolism, Department of Internal Medicine, Taichung Veterans General Hospital, Taichung, 40705 Taiwan; 9 Institute of Clinical Medicine, School of Medicine, National Yang Ming Chiao Tung University, Taipei, 11221 Taiwan

**Keywords:** Palliative care, Hospice care, End-of-life, Place of death

## Abstract

**Background:**

Dying at home accompanied by loved-ones is regarded favorably and brings good luck in Taiwan. This study aimed to examine the relevant factors affecting whether an individual dies at home or not in a group of terminal patients receiving palliative home care service.

**Methods:**

The patients who were admitted to a palliative home care service at a hospital-affiliated home health care agency were consecutively enrolled between March 1, 2021 and March 31, 2022. During the period of care, the instruments of the palliative care outcomes collaboration was used to assess patients in each home visit twice a week, including symptom assessment scale, palliative care problem severity score, Australia-modified Karnofsky performance status, resource utilization groups-activities of daily living, and palliative care phase.

**Results:**

There were 56 participants (53.6% female) with a median age of 73.0 years (interquartile range (IQR) 61.3–80.3 y/o), of whom 51 (91.1%) patients were diagnosed with cancer and 49 (96.1%) had metastasis. The number of home visits was 3.5 (IQR 2.0–5.0) and the average number of days under palliative home care service was 31 (IQR 16.3–51.5) before their death. After the end of the study, there was a significant deterioration of sleeping, appetite, and breathing problems in the home-death group, and appetite problems in the non-home death patients. However, physician-reported psychological/spiritual problems improved in the home-death group, and pain improved in the non-home death patients. Physical performance deteriorated in both groups, and more resource utilization of palliative care was needed. The 44 patients who died at home had greater cancer disease severity, fewer admissions, and the proportion of families desiring a home death for the patient was higher.

**Conclusions:**

Although the differences in palliative outcome indicators were minor between patients who died at home and those who died in the hospital, understanding the determinants and change of indicators after palliative care service at different death places may be helpful for improving the quality of end-of-life care.

## Highlights

• Hospice care is a type of palliative care that focuses on patients with advanced, life-limiting illnesses, and these terms are sometimes used interchangeably for patients who are in the last months or years of their life.

• The number of patients finally dying at home was high in our palliative home service, and the congruence regarding place of death between patients and family, their preferred place of death, cancer with metastasis, and the hospital admission during palliative home care were associated with the actual place of death.

• Psychological well-being and spirituality was more enhanced in home-death patients, and pain was improved in hospital-death patients.

• Understanding the determinants and change of indicators during palliative care service at different death places may be helpful for improving the quality of end-of-life care.

## Introduction

Palliative care is holistic care, encompassing all aspects of patients’ physical, psychological, social, and spiritual needs, as well as the needs of their caregiver, and includes relieving patients and their families of suffering [1]. Hospice care is a type of palliative care that focuses on patients with advanced, life-limiting illnesses, and the term hospice care is sometimes used interchangeably with palliative care for patients who are in the last months or years of their life [1, 2]. Generally, place of death is regarded as a key policy marker of end-of-life care success worldwide [3–5]. Supporting patients at the end of life to enable them to die at home, rather than being admitted to hospital is a significant task for community nursing teams [5]. In Taiwan, palliative care programs have been implemented in Taiwan’s National Health Insurance (NHI) system since 1996, and includes palliative home care, palliative inpatient care, and palliative care consultation service [6]. According to traditional culture in Taiwan, dying at home and being cared for by family members is regarded favorably and brings good luck [7–9]. Compared with dying in a hospital, dying at home is often considered the most appropriate and ideal place for many people [9].

The palliative care outcomes collaboration (PCOC) was established and funded by Australia’s Department of Health in 2005. It employs standardized validated clinical assessment tools to identify and measure the impact of palliative service delivery for people with a life-limiting illness, their families, and caregivers [10–23]. The PCOC instruments, which are composed of symptom assessment scale (SAS) [10–23], palliative care problem severity score (PCPSS) [12, 13, 17, 20, 21], Australia-modified karnofsky performance status (AKPS) [14, 19–21, 23], resource utilisation groups-activities of daily living (RUG-ADL) [10, 20, 21], and palliative care phase (PCP) [12, 13, 17, 19–21], are widely used for the evaluation of patients’ conditions, and for planning of interventions in palliative care. Several studies have demonstrated that these instruments are feasible and useful in systematically improving outcomes in individuals receiving palliative care at local, subnational, and national levels [17–23]. However, in Taiwan, there are few data on PCOC for patients in palliative care programs. The study aimed to examine the relevant factors affecting whether an individual dies at home or not in a group of terminal patients receiving palliative home care service. The instruments of PCOC were regularly used to evaluate patients during palliative home care service, and the changes in the respective scores (e.g. symptom burden, palliative care phase, functional status) were examined. Also, their relevance in relation to death place was also determined.

## Methods

### Study design

This study was conducted at a hospital-affiliated home health care agency in a medical center in Taiwan between March 1, 2021 and March 31, 2022. During the study period, the patients with an advanced disease in the terminal stages who were referred to palliative home service care were consecutively enrolled. A multidisciplinary team comprising nurses, doctors, and social workers provided comprehensive palliative care services addressing the patients’ physical, psychosocial and spiritual needs at home regularly and as necessary. According to the regulations, a professional nurse could visit twice a week and the assessment was performed approximately every three days. Nevertheless, during palliative home care service, admission to a hospice ward could be arranged according to the patient's condition and request. The study was approved by the Institutional Review Board of the medical center (IRB no: CE22109A) and all methods were carried out in accordance with the approved study protocol under the standard regulations and the Declaration of Helsinki.

### Participants

**Fig. 1:**
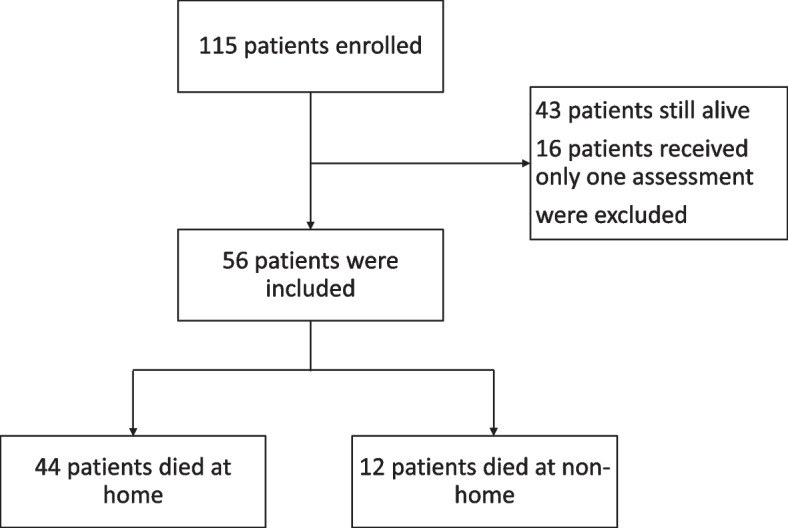
Flowchart of patients who received palliative home care services

The inclusion criteria were patients with an advanced disease with terminal stage requiring a diagnosis by two physicians according to the Hospice Palliative Care Act, which was promulgated by Taiwan’s Ministry of Health and Welfare. Moreover, at the beginning of the patients received palliative home care service, they were first required to undergo at least two evaluations of PCOC instruments to determine their trajectory. The exclusion criteria were patients or their family/caregiver refused to be assessed by PCOC instruments, or they could not complete the whole study period for any reason. Initially, 115 patients were enrolled in the study from March 1, 2021 and March 31, 2022. Of these, 59 patients were not eligible, including 43 patients still alive at the end of the study and 16 patients who were just evaluated once using PCOC instruments during the study period. Finally, 56 patients had died at the time of analysis (Fig. 1).

### Outcome and assessment procedures

The primary outcome was the place of death (i.e. home (nursing home) and non-home (hospital)). Basic personal information and medical records of all enrolled patients were reviewed, including age, gender, disease diagnosis, medications, disease duration, comorbidities, the numbers of admissions (the last time of admission was excluded, if dying in the hospital), living situation (with family, or non-family/alone), and financial subsidy (NHI premium alone, or others: disability or veteran). Tube dependency was also investigated, including nasogastric tubes, foley catheter, tracheostomy, nephrostomy, or cystostomy tubes.

Each of the PCOC instrument was assessed by a skilled visiting nurse for palliative care in the patient’s home only and was not performed in a hospital admission during the study period [10–23]. The SAS of PCOC instruments is a patient-reported scale used to measure eight dimensions: pain, insomnia, nausea, bowel problems, appetite problems, breathing problems, fatigue, and others. The score range is from 0 to 10, with higher values indicating more severe distress [10–23]. Among the PCOC instruments, PCPSS is a clinician-reported scale used to measure four palliative care domains: pain, other symptoms, psychological/spiritual, and family/carer problems. The score range is from 0 to 3, with higher values indicating worse problem severity [12, 13, 17, 20, 21]. Another PCOC instrument, the AKPS, is a clinician-observed scale used to measure the patient’s overall performance status, such as common tasks relating to activity, work, and self-care. The score range is from 0 to 100, with higher values indicating better physical abilities [14, 19–21, 23]. The RUG-ADL (a PCOC instrument) is another clinician-reported scale used to measure basic performance status, such as bed mobility, toileting, transfers, and eating. The score range is from 4 to 18, with higher values indicating poorer performance and need of more assistance [10, 20, 21]. The PCP (a PCOC instrument) is a measure of relative resource utilisation linked directly to clinical needs, which consists of five phases: stable, unstable, deteriorating, terminal, and bereavement. Whenever the clinical situation changed, the patient is reassessed and the care plan is modified [12, 13, 17, 19–21].

### Statistical analyses

Continuous variables were expressed as median and interquartile range (IQR, 25%-75%). Categorical data were expressed as number and percentage. The significance of the difference between groups was assessed using the Mann–Whitney U test (medians, non-Gaussian populations), and Pearson’s chi-squared or Fisher’s exact test (proportions). Paired comparisons were made using the Wilcoxon signed rank test or Friedman test for continuous variables, and McNemar’s or Cochran’s Q tests for categorical variables during follow-up. Statistical analyses were performed using SPSS version 22.0 (SPSS Inc., Chicago, IL, USA). Statistical significance was set at *p* < 0.05.

## Results

The characteristics of the study population are shown in Table 1. In total, the median age was 73.0 (IQR 61.3–80.3) years old with males accounting for 46.4% of the patients and females 53.6%. Fifty-one (91.1%) patients were diagnosed with cancer and 49 (96.1%) had metastasis. The number of patients finally dying at home was 44 (78.6%) in our palliative home service, and during palliative home care service, the median number of home visits was 3.5 (IQR 2.0–5.0) and the number of days under palliative home care service was 31 (IQR 16.3–51.5) days before death. Forty-four (78.6%) patients were alert, 46 (82.1%) patients did not need oxygen therapy, and 39 (69.6%) patients could eat by mouth. Eighteen (32.1%) patients had incontinence, 9 (16.1%) patients required tube/catheter change, and 6 (10.7%) patients had wound issues. Fifty-one (91.1%) patients lived with their family and only 9 (16.1%) patients had other financial subsidies. Almost all patients and their respective families were in agreement about the place of death. Seven (12.5%) patients were admitted to hospital while under palliative home care service. The patients who died at home had greater severity of cancer disease, fewer admissions, and the proportion of families desiring a home-death was higher (Table 1).

**Table 1:** Baseline characteristics of the participants

Demographic characteristics	Total (*n* = 56)		Place of death				*p* value
			**Home (** ***n*** ** = 44)**		**Non-Home (** ***n*** ** = 12)**		
Age (years)	73	(61.3–80.3)	73.0	(62.0–81.0)	74.5	(59.5–77.8)	0.952
Sex, n(%)							1.000
Male	26	(46.4%)	20	(45.5%)	6	(50.0%)	
Female	30	(53.6%)	24	(54.5%)	6	(50.0%)	
Cancer	51	(91.1%)	40	(90.9%)	11	(91.7%)	1.000
Metastasis	49	(96.1%)	40	(100.0%)	9	(81.8%)	0.043
Home visit (times)	3.5	(2.0–5.0)	3.0	(3.0–5.0)	4.0	(2.0–4.8)	0.806
Palliative care (days)	31	(16.3–51.5)	30.5	(16.3–51.5)	34.0	(15.8–59.3)	0.668
Level of consciousness							0.155
Alert	44	(78.6%)	36	(81.8%)	8	(66.7%)	
Lethargy	8	(14.3%)	4	(9.1%)	4	(33.3%)	
Obtundation	3	(5.4%)	3	(6.8%)	0	(0%)	
Stupor	0	(0%)	0	(0%)	0	(0%)	
Coma	1	(1.8%)	1	(2.3%)	0	(0%)	
Oxygen demand							0.196
Room air	46	(82.1%)	38	(86.4%)	8	(66.7%)	
Oxygen therapy	10	(17.9%)	6	(13.6%)	4	(33.3%)	
Feeding							0.738
Oral	39	(69.6%)	31	(70.5%)	8	(66.7%)	
Nasogastric tube	9	(16.1%)	6	(13.6%)	3	(25.0%)	
Nasojejunal tube	1	(1.8%)	1	(2.3%)	0	(0%)	
Peripheral parenteral nutrition	7	(12.5%)	6	(13.6%)	1	(8.3%)	
Incontinence	18	(32.1%)	15	(34.1%)	3	(25.0%)	0.732
Enterostomy	4	(7.1%)	4	(9.1%)	0	(0%)	0.567
Percutaneous nephrostomy	1	(1.8%)	1	(2.3%)	0	(0%)	1.000
Foley catheter	8	(14.3%)	7	(15.9%)	1	(8.3%)	0.672
Wound care	6	(10.7%)	5	(11.4%)	1	(8.3%)	1.000
Living situation							0.574
Family	51	(91.1%)	39	(88.6%)	12	(100.0%)	
Non-family/alone	5	(8.9%)	5	(11.4%)	0	(0%)	
Financial subsidy							0.666
National Health Insurance premium alone	47	(83.9%)	36	(81.8%)	11	(91.7%)	
Still had other (disability or veteran)	9	(16.1%)	8	(18.2%)	1	(8.3%)	
Patient hope of place of death							< 0.001
Home	46	(82.1%)	43	(97.7%)	3	(25.0%)	
Non-Home	10	(17.9%)	1	(2.3%)	9	(75.0%)	
Family hope of place of death							< 0.001
Home	45	(80.4%)	42	(95.5%)	3	(25.0%)	
Non-Home	11	(19.6%)	2	(4.5%)	9	(75.0%)	
The numbers of hospice ward admission							0.032
0	49	(87.5%)	41	(93.2%)	8	(66.7%)	
1	7	(12.5%)	3	(6.8%)	4	(33.3%)	

At baseline, SAS showed fatigue was the most bothersome symptom among all patients. Almost all patients had poorer performance status according to AKPS and RUG-ADL, as they were at the unstable phase. Psychological/spiritual problem was significantly worse in the home-death patients than non-home death patients (Table 2). At the last assessment, SAS showed difficulty sleeping, appetite problems and fatigue were the three major burdens among all patients. The total performance status got worse in all patients. There was almost no difference in any parameters of SAS and PCPSS between the two groups (Table 3). However, sleeping, appetite, and breathing problems, and physical performance changed more in the home-death group, whereas appetite problem and pain were found in the non-home death patients (Table 4).

**Table 2:** **T**he first assessment in each group

	**Total**		**Home**		**Non-Home**		***p*** value
	**Median**	**IQR**	**Median**	**IQR**	**Median**	**IQR**	
SAS							
Difficulty sleeping	1	(0–6)	1	(0–6)	2	(0–5)	0.579
Appetite problems	1	(0–4)	2	(0–4)	0.5	(0–4.5)	0.384
Nausea	0	(0–2)	0	(0–2)	0	(0–2.75)	0.657
Bowel problems	1	(0–5)	1	(0–5)	0.5	(0–5)	0.670
Breathing problems	0	(0–4)	0	(0–4)	1	(0–4.5)	0.424
Fatigue	2	(1–7)	1	(1–7)	3	(0.25–7.5)	0.811
Pain	1	(0–5)	1	(0–5)	1	(0.25–5.75)	0.900
PCPSS							
Pain	1	(0–2)	1	(0–2)	1	(1–1.75)	0.801
Other symptoms	1	(1–2)	1	(1–2)	1	(0–1.75)	0.070
Psychological/spiritual	1	(1–1)	1	(1–2)	1	(0–1)	0.041
Family/carer	1	(1–2)	1	(1–2)	1	(0.25–1.75)	0.341
AKPS	40	(30–40)	40	(30–40)	40	(30–50)	0.698
RUG-ADL							
Bed mobility	3.5	(3–4)	4	(3–4)	3	(3–5)	0.783
Toileting	4	(3–4)	4	(3–4)	4	(1.5–5)	0.958
Transfer	4	(3–4)	4	(3–4)	3.5	(1.5–4.75)	0.950
Eating	2	(2–3)	2	(2–3)	2	(1–3)	0.392
Palliative care phase, n(%)							0.527
1	19	(26.39%)	14	(24.14%)	5	(35.71%)	
2	36	(50.00%)	28	(48.28%)	8	(57.14%)	
3	11	(15.28%)	10	(17.24%)	1	(7.14%)	
4	6	(8.33%)	6	(10.34%)	0	(0.00%)

**Table 3:** The last assessment in each group

	**Total**		**Home**		**Non-Home**		***p*** value
	**Median**	**IQR**	**Median**	**IQR**	**Median**	**IQR**	
SAS							
Difficulty sleeping	3	(0–8)	3	(0–8)	1.5	(0–8.75)	0.764
Appetite problems	3	(0–9)	2	(0–8)	3	(0–10)	0.539
Nausea	0	(0–2)	0	(0–2)	0	(0–5.25)	0.874
Bowel problems	0	(0–5)	1	(0–5)	0	(0–8)	0.956
Breathing problems	2	(0–7)	2	(0–7)	4	(1–7.5)	0.391
Fatigue	3	(1–8)	3	(1–8)	2.5	(0.25–9.75)	0.918
Pain	2	(0–6)	2	(0–6)	1	(0.25–6.5)	0.656
PCPSS							
Pain	1	(0–1)	1	(0–1)	0.5	(0–1)	0.247
Other symptoms	1	(1–2)	1	(1–2.75)	1	(0.25–1.75)	0.397
Psychological/spiritual	1	(0–1)	1	(0–1)	1	(0–1.75)	0.818
Family/carer	1	(1–1)	1	(1–1)	1	(1–2)	0.218
AKPS	20	(20–30)	20	(20–30)	20	(20–37.5)	0.655
RUG-ADL							
Bed mobility	4	(4–5)	4	(4–4.75)	4	(3.25–5)	0.675
Toileting	4	(4–5)	4	(4–5)	4.5	(3.25–5)	0.706
Transfer	4	(4–5)	4	(4–5)	4	(3.25–5)	0.802
Eating	3	(3–3)	3	(3–3)	3	(2–3)	0.141
Palliative care phase, n(%)							1.000
1	7	(12.50%)	6	(13.64%)	1	(8.33%)	
2	12	(21.43%)	9	(20.45%)	3	(25.00%)	
3	9	(16.07%)	7	(15.91%)	2	(16.67%)	
4	28	(50.00%)	22	(50.00%)	6	(50.00%)

**Table 4:** The difference between the first and the last assessments in each group

	**Home**					**Non-Home**				
	**The first data**		**The last data**			**The first data**		**The last data**		
	**Median**	**IQR**	**Median**	**IQR**	***p*** ** value**	**Median**	**IQR**	**Median**	**IQR**	***p*** ** value**
SAS										
Difficulty sleeping	1	(0–6)	3	(0–8)	0.037	2	(0–5)	1.5	(0–8.75)	0.078
Appetite problems	2	(0–4)	2	(0–8)	0.040	0.5	(0–4.5)	3	(0–10)	0.017
Nausea	0	(0–2)	0	(0–2)	0.421	0	(0–2.75)	0	(0–5.25)	0.465
Bowel problems	1	(0–5)	1	(0–5)	0.883	0.5	(0–5)	0	(0–8)	0.394
Breathing problems	0	(0–4)	2	(0–7)	0.029	1	(0–4.5)	4	(1–7.5)	0.061
Fatigue	1	(1–7)	3	(1–8)	0.142	3	(0.25–7.5)	2.5	(0.25–9.75)	0.307
Pain	1	(0–5)	2	(0–6)	0.536	1	(0.25–5.75)	1	(0.25–6.5)	0.551
PCPSS										
Pain	1	(0–2)	1	(0–1)	0.239	1	(1–1.75)	0.5	(0–1)	0.014
Other symptoms	1	(1–2)	1	(1–2.75)	0.726	1	(0–1.75)	1	(0.25–1.75)	0.271
Psychological/spiritual	1	(1–2)	1	(0–1)	0.006	1	(0–1)	1	(0–1.75)	0.480
Family/carer	1	(1–2)	1	(1–1)	0.058	1	(0.25–1.75)	1	(1–2)	0.317
AKPS	40	(30–40)	20	(20–30)	< 0.001	40	(30–50)	20	(20–37.5)	0.023
RUG-ADL										
Bed mobility	4	(3–4)	4	(4–4.75)	< 0.001	3	(3–5)	4	(3.25–5)	0.071
Toileting	4	(3–4)	4	(4–5)	< 0.001	4	(1.5–5)	4.5	(3.25–5)	0.102
Transfer	4	(3–4)	4	(4–5)	< 0.001	3.5	(1.5–4.75)	4	(3.25–5)	0.058
Eating	2	(2–3)	3	(3–3)	< 0.001	2	(1–3)	3	(2–3)	0.063
Palliative care phase, n(%)					< 0.001					0.016
1–2	37	(84.1%)	15	(34.1%)		11	(91.7%)	4	(33.3%)	
3–5	7	(15.9%)	29	(65.9%)		1	(8.3%)	8	(66.7%)	

## Discussion

In this study, the instruments of the palliative care outcomes collaboration were assessed by a hospital-affiliated home health care agency, and the results revealed that congruence between patients and family, their preferred place of death, cancer with metastasis, and the once hospitalization were all associated with the decision of death place. Although several patient-reported problems deteriorated during palliative home service in patients that desired a home death, their psychological well-being and spirituality was still slightly improved. However, deteriorated sleeping, appetite, and breathing problems, and physical performance status required special care and assistance.

Some previous studies in Taiwan showed the percentage of cancer patients who died at home was around 35.7% to 32.4% from 2001 to 2006 [24], while another report revealed the rate of a home death under palliative care home service was 43.6% [25]. Physician home visits increased the likelihood of a home death, but previous hospitalizations within one year decreased the likelihood of a home death [25]. It is generally thought that the professional environment and staff of a hospital can provide superior inpatient care in comparison to home care [26]. However, if the motivation and requirements of dying patients at the hospital can be understood, an optimal solution can be planned to increase patient and family satisfaction, and if their preferred place of care is the home, carers can make arrangements to provide better care so that the patient can die well [26, 27]. A systematic review demonstrated functional impairment, patients’ preferred place of death, high home care intensity, and strong family relationship increased the likelihood of dying at home among cancer patients [28]. In line with a previous study, our study showed 51 (91.1%) patients were diagnosed with cancer. Moreover, we found cancer severity was a determinant factor in early recognition and management of impending death. It has been proposed that in non-cancer diseases, the uncertain disease course might give patients inappropriate expectations about length of life [29].

Based on a literature review, in patients who preferred dying at home pain was less of a problem, but the prevalence of dependent functional status was higher [30]. Furthermore, those who knew their treatment and prognosis were more likely to choose the home as their preferred place of death [30]. Our study supports previous findings that patients dying at home show poorer performance and need more assistance. Patients with life-limiting illnesses often suffer from multiple discomfort symptoms that reduce quality of life [23], including breathlessness, bowel problems, fatigue, and pain [18, 22, 31]. It has been reported that fatigue was a common symptom although it is underrecognized frequently [28], and one third of patients may experience significant breathlessness [29], or suffer from at least some degree of bowel problems [30]. Hence, management of all these discomforts is a priority task at the end of life, while choice of preferred place of death, ensuring contact with family/friends, addressing spiritual needs, and other non-medical concerns were sometimes ranked lower [32]. In addition, symptom control at home was more challenging than in the hospital, which indicates the palliative home care team could be more aggressive toward palliative symptom control. In our study, we found that symptoms had not changed over time either in home or non-home death patients because, despite their possible increase, they had been well controlled, and thus this affected the actual place of death.

Psychological factors may influence the preferred place of death among cancer patients, such as patients' level of anxiety, and awareness of the burden on family and caregivers [30]. Our study found that in home-death patients, their wellbeing was worse than in the non-home death group at first, however they could be significantly improved finally even though dying at home may bring more challenges in terms of symptom control, and preferences about place of death may change over time [27].

A systematic review demonstrated that patients’ preferences for place of death are complex [33], and depend on substantial input from family and professional community nursing teams. In addition, recent studies have challenged the priority given to location in end-of-life care [34]. There was no clear relation between the symptoms and care services [20]. In our study, PCP was not different between the two groups, which might be attributable to the fact that our care teams could provide a rapid response service instantly when necessary. Apart from professional support, advanced care planning was shown to increase the chances of dying at home and improve quality of care [35].

To provide better quality of palliative care, adequate policies and guidelines, continuity and coordination of care, and better knowledge and skills for all caregivers were needed [36]. It has been shown that early palliative care for patients with advanced disease had better outcomes in quality of life, not only for patients, but also for the family/caregivers [37–40].

The study had some limitations. First, the findings were based on data from a single hospital-affiliated home health care agency and the study sample size was small so the findings may not be generalizable to other populations. Second, the assessment of our participants who were under palliative home care service was not done at a fixed interval so there might be potential selection bias. Third, we did not conduct laboratory examinations, such as testing of blood samples, and these factors may have had a significant impact on hospital admission.

## Conclusion

This study found that the number of patients finally dying at home was higher in our palliative home service in comparison with previous studies in Taiwan. The patients’ preferred place of death, congruence between patients’ and families preferred place of death, cancer with metastasis, and hospital admissions during palliative care were associated with the actual place of death. Dying at home could promote psychological well-being and spirituality, but could bring more challenges with respect to symptom relief, including sleeping, appetite, and breathing problems. Understanding the relevant determinants and change of indicators after palliative care service at different death places may be helpful for improving end-of-life care and may help patients die well.

## Data Availability

The datasets used and analyzed during the current study are available from the corresponding author on reasonable request.

## Abbreviations

AKPS: Australia-modified karnofsky performance status
IQR: Interquartile range
NHI: National Health Insurance
PCOC: Palliative care outcomes collaboration
PCP: Palliative care phase
PCPSS: Palliative care problem severity score
RUG-ADL: Resource utilisation groups-activities of daily living
SAS: Symptom assessment scale
